# P78 Implementation of a penicillin allergy de-labelling policy at a tertiary hospital

**DOI:** 10.1093/jacamr/dlaf230.085

**Published:** 2025-12-04

**Authors:** Noor Anjum

**Affiliations:** Liverpool Heart and Chest Hospital, Liverpool, UK

## Abstract

**Background:**

Following national antimicrobial stewardship initiatives highlighting the risks associated with penicillin allergy labels s well as BSACI guidelines on the provision of a penicillin allergy de-labelling service aimed at clinicians who are not trained in immunology or allergy, a penicillin allergy de-labelling policy was needed for the evaluation and testing of patients at our Trust with unverified labels of penicillin allergies. This provides a framework for administering a non-specialist drug provocation challenge test to safely establish whether a patient has a true penicillin allergy or not. The evidence behind why spurious penicillin allergy labels should be investigated and removed includes the fact that when formally assessed, over 90% of patients labelled as allergic are in fact not. Penicillin allergy labels are associated with increased healthcare costs, length of inpatient stays, and patient mortality. A penicillin allergy diagnosis increases the risk of MRSA, C Difficile and VRE infections and also leads to higher use of WHO Watch and Reserve listed antibiotics which drives antimicrobial resistance. Ultimately, the de-labelling service is a key strategy for improving patient outcomes and antimicrobial stewardship at LHCH.

**Objectives:**

To implement a successful penicillin allergy de-labelling protocol for administering a test dose of penicillin in patients, without requiring input from an allergist or immunologist; to write an evidence-based penicillin allergy de-labelling protocol; to educate clinicians on how to conduct a penicillin allergy de-labelling drug provocation challenge test; and to develop electronic prescribing tools to support documentation, prescription and administration of penicillin allergy de-labelling drug provocation challenge test.

**Methods:**

A penicillin allergy de-labelling protocol was published by the lead antimicrobial pharmacist, which the microbiology team promoted and directed clinicians towards whilst conducting microbiology ward rounds three times a week. From our electronic prescribing system, data on the use of the penicillin allergy de-labelling order set was ran by our internal audit team to establish how many patients were de-labelled during the year of 2024. The medication charts were reviewed to establish what the outcomes of the test dose were and whether penicillin-based treatment was utilized. The penicillin allergy records of patients who were based within the Cheshire and Merseyside ICB were reviewed by a primary care pharmacist.

**Standards:**

How many penicillin provocation challenge test doses were prescribed using the electronic prescribing order set versus without. How many patients were successfully de-labelled as an inpatient. How many patients were treated with penicillin-based antibiotics as an inpatient, following the de-labelling drug provocation challenge test. How many patients were successfully de-labelled on discharge by their primary care team (in the Cheshire and Merseyside ICB).

**Results:**

A total of 11 patients were successfully de-labelled as an inpatient in 2024, and of the 4 patients under the Cheshire and Merseyside ICB, only 1 patient was successfully de-labelled on their primary care records.

**Conclusions:**

The de-labelling protocol and order set has been a success within our Trust, leading to optimal antibiotic regimens, however this is not being actioned within the community to improve future outcomes for patients.

**Recommendations:**

(i) The communication between the Trust, patient and GP surgeries to be strengthened with the use of the newly established ‘NHS C&M Penicillin Allergy De-labelling Communication Form’. (ii) A Trust-wide staff engagement project to establish prescribing habits and confidence around penicillin allergy de-labelling.

